# Designing injectable dermal matrix hydrogel combined with silver nanoparticles for methicillin-resistant Staphylococcus aureus infected wounds healing

**DOI:** 10.1186/s40580-024-00447-0

**Published:** 2024-10-17

**Authors:** Sunfang Chen, Jun Yao, Shicheng Huo, Chennan Xu, Ruting Yang, Danhua Tao, Bin Fang, Gaoxiang Ma, Zaihua Zhu, Ye Zhang, JingJing Guo

**Affiliations:** 1 Department of Orthopedic Surgery, Spine Center, the Central Hospital Affiliated to Shaoxing University, Shaoxing, 321030 China; 2 Department of Orthopedic Surgery, Spine Center, Changzheng Hospital, Navy Medical University, Shanghai, 200003 China; 3 Department of Pathology, the Central Hospital Affiliated to Shaoxing University, Shaoxing, 321030 China; 4 Division of Rheumatology and Immunology, Huashan Hospital, Fudan University, Shanghai, 200040 China; 5 Department of Pharmacy, the Central Hospital Affiliated to Shaoxing University, Shaoxing, 321030 China

**Keywords:** Injectable dermal matrix, Silver nanoparticles, MRSA, Immune environmentalism, Wound healing

## Abstract

Hydrogel-based delivery systems have now emerged as a pivotal platform for addressing chronic tissue defects, leveraging their innate capacity to suppress pathogenic infections and facilitate expedited tissue regeneration. In this work, an injectable hydrogel dressing, termed AgNPs-dermal matrix hydrogel (Ag@ADMH), has been designed to expedite the healing process of wounds afflicted with methicillin-resistant Staphylococcus aureus (MRSA), featuring sustained antibacterial efficacy. The synthesis of the hydrogel dressing entailed a self-assembly process of collagen fibers within an acellular dermal matrix to construct a three-dimensional scaffold, encapsulated with plant polyphenol-functionalized silver nanoparticles (AgNPs). The Ag@ADMH demonstrated exceptional biocompatibility, and enables a sustained release of AgNPs, ensuring prolonged antimicrobial activity. Moreover, the in vitro RT-qPCR analysis revealed that compared with ADMH, Ag@ADMH diminish the expression of iNOS while augmenting CD206 expression, thereby mitigating the inflammatory response and fostering wound healing. Especially, the Ag@ADMH facilitated a reduction in M1 macrophage polarization, as evidenced by a significant decrement in the M1 polarization trend and an enhanced M2/M1 ratio in dermal matrix hydrogels laden with AgNPs, corroborated by confocal microscopy and flow cytometry analyses of macrophage phenotypes. The in vivo assessments indicated that Ag@ADMH minimized fibrous capsule formation. In a full-thickness skin defect model of MRSA infection, the formulation significantly attenuated the inflammatory response by reducing MPO and CD68 expression levels, concurrently promoting collagen synthesis and CD34 expression, pivotal for vasculogenesis, thereby accelerating the resolution of MRSA-infected wounds. These attributes underscore the injectable extracellular matrix hydrogel as a formidable strategy for the remediation and regeneration of infected wounds.

**Graphical Abstract:**

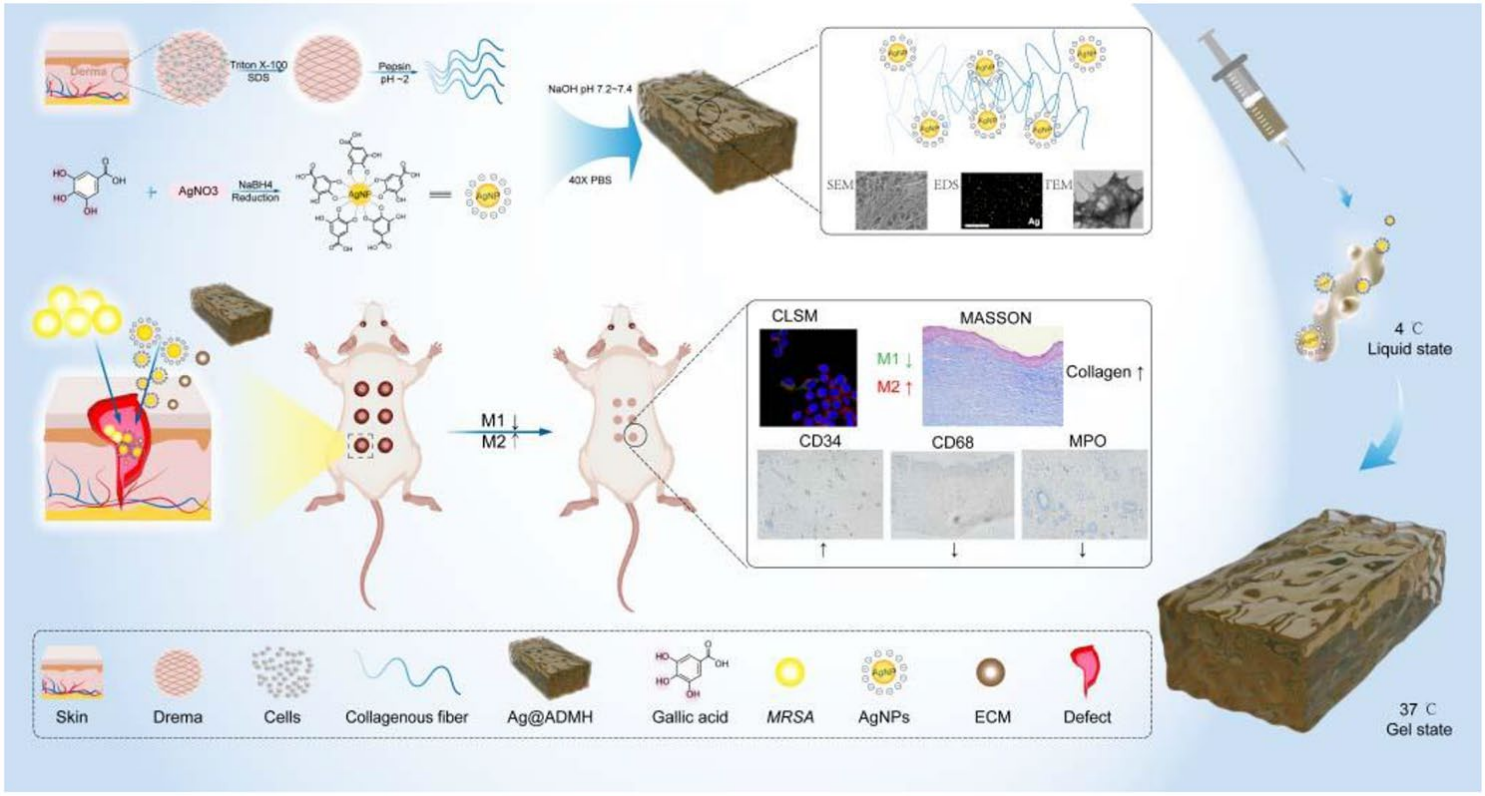

**Supplementary Information:**

The online version contains supplementary material available at 10.1186/s40580-024-00447-0.

## Introduction

The epidermis, functioning as a primary barrier [1], represents the most extensive organ within the human anatomy. Upon the event of an injury, epidermal lesions become susceptible to microbial invasions due to a compromise in the innate protective mechanisms of the skin, potentially obstructing the healing trajectory [2, 3]. Microbial invasions, notably bacterial, are frequently associated with the induction of chronic inflammation [4], critically impeding the healing continuum—spanning from granulation to the phases of proliferation and tissue remodeling. This disruption often culminates in the protracted recovery or stagnation of chronic ulcerations [5]. Although the introduction of antimicrobial agents has mitigated the escalation of bacterial infections to a degree, the proliferation of antimicrobial resistance—fueled by excessive antibiotic utilization—has fostered the prevalence of resistant pathogens within both community and hospital settings [6]. The management of infectious sequelae post-trauma, particularly the amelioration of chronic wounds instigated by methicillin-resistant Staphylococcus aureus (MRSA), remains a pressing clinical challenge [7].

The primary objective of conventional wound coverings, including gauze, cotton wool, and bandages, is centered on maintaining moisture at the lesion site and managing wound exudates [8, 9]. Despite their ubiquity, these modalities fall short in addressing severe traumas and persistent ulcers due to their dependence on gradual and passive recuperation processes, alongside a lack of prophylaxis against microbial invasions. In recent advancements, hydrogels, characterized by their hydration-rich and three-dimensional (3D) frameworks, have gained prominence as substrates for fostering a damp and permeable milieu conducive to the proliferation and integration of reparative cells, establishing sustained-release mechanisms for therapeutic agents, or enhancing stem cell therapies to expedite the repair of dermal damage [10–12]. Nonetheless, these approaches are frequently curtailed by manufacturing intricacies, over-elaborate compositions, potential drug-induced toxicities, or elevated expenditures, posing substantial barriers to their clinical deployment [5]. Intriguingly, bioactive materials have demonstrated efficacy in modulating the biological activities of host cells [13–16], with these biomaterials or frameworks often providing the ideal structural and functional microenvironments for tissue regeneration, thus emerging as a novel avenue in regenerative medicine. The Extracellular Matrix (ECM), a biodegradable construct imbued with an array of bioactive compounds, plays a crucial role in immune modulation, angiogenesis, and collagen synthesis [17]. ECM derived from identical sources is particularly beneficial for the repair of like tissues. For instance, acellular spongy bone markedly enhances cranial regeneration in rodents [14], while acellular periosteum not only facilitates osseous renewal but also ameliorates the local inflammatory landscape and stimulates blood vessel formation [13]. Leveraging these merits, ECM frameworks are deemed optimal for cutaneous wound closure. Predominantly, ECMs are known to promote the M2 polarization of macrophages. For example, acellular ECM frameworks in the mucosal linings of the bladder, esophagus, colon, and small intestine can induce a shift towards the M2 phenotype in macrophages [18]. Although numerous investigations on the dermal ECM have highlighted its therapeutic potential [19], evidence suggests its significant role in encouraging the M1 polarization of macrophages and elevating inflammatory cytokine levels [20]. An excessively inflammatory state can obstruct the healing process, potentially restricting its broader application.

Silver nanoparticles (AgNPs) have garnered extensive attention for their potential to mitigate bacterial infections, attributed to their superior antimicrobial efficacy, straightforward synthesis, difficulty in engendering drug-resistant bacterial strains, and broad-spectrum antibacterial capabilities [21]. Notably, AgNPs synthesized via plant polyphenols, such as gallic acid-derived silver nanoparticles, have been demonstrated to facilitate controlled release mechanisms, sustain local AgNP concentrations within a safe and efficacious threshold, and exert anti-inflammatory effects [22–24], notably diminishing the expression of IL-1B, iNOS, and other inflammatory mediators. Despite the significant advancements in utilizing AgNPs for bacterial infection management and inflammation mitigation, the direct application of nano-silver in wound care remains less than optimal, particularly in addressing complex and extensive injuries. Concurrently, elevated concentrations of nano-silver have been associated with notable cytotoxicity [25]. Consequently, there persists a pronounced demand for wound dressings capable of gradually releasing silver nanoparticles to optimize therapeutic outcomes while minimizing potential risks.

In the realm of chronic dermal wound management, the development of a multifunctional dressing capable of modulating the wound microenvironment—encompassing inflammatory response and angiogenesis—while concurrently eradicating bacterial infections, is crucial to facilitate the intricate and sequential process of skin regeneration [26]. To this end, we have incorporated silver nanoparticles with plant polyphenol constituents, such as gallic acid-enhanced silver nanoparticles. This combination has demonstrated that gallic acid can augment the M2 macrophage polarization [27], while silver nanoparticles contribute to robust antimicrobial activity and anti-inflammatory effects. A novel hydrogel formulation (Ag@ADMH) has been devised by encapsulating gallic acid-modified silver nanoparticles within a dermal matrix hydrogel. This innovative approach, through the gradual release of plant polyphenol silver nanoparticles, aims to proactively modulate pro-inflammatory mechanisms within the dermal ECM and prevent overwhelming inflammation. Such a strategy could represent an optimal solution for addressing the challenges associated with chronic skin wound healing.

In this context, we have designed an injectable hydrogel dressing (Ag@ADMH), marked by its exceptional antimicrobial prowess and capability to regulate the immune microenvironment, aimed at expediting the recovery of infected wounds through the application of plant polyphenol-coated silver nanoparticles within a dermal extracellular matrix hydrogel. The resultant hydrogels exhibit superior injectable properties, enabling seamless adaptation to wounds of any configuration. Furthermore, these hydrogels are engineered to facilitate the transition of macrophages from M1 to M2 polarization via self-degradation and the release of silver nanoparticles. Moreover, the synergy with the extracellular matrix of the same origin significantly enhances the repair of analogous tissues, with the dermal ECM specifically promoting collagen synthesis and angiogenesis. Consequently, the innovatively crafted antibacterial hydrogel dressing harbors significant promise for clinical utility in managing infected wounds.

## Experimental methods

### Materials

Triton-X100, SDS, NaOH, HCl, pepsin, trypsin, phosphate buffer saline (PBS), NaBH4, AgNO_3_, and Gallic Acid, among others, were acquired from Shanghai Aladdin Bio-Chem Technology Co., Ltd. The DNA extraction kit was sourced from Beijing Kangwei Century Biotech Co., Ltd., while the hydroxyproline (HYP) assay kit was procured from Beijing Soleibao Technology Co., Ltd.

### Dermal matrix isolation

The dermal matrix derived from domestic swine (aged 6 months) was procured from a local abattoir. The procurement process entailed the meticulous removal of subcutaneous tissue under aseptic conditions, ensuring the retention of the dermis and epidermis layers. To isolate a pristine dermal matrix, the initially sectioned skin tissue underwent cryogenic lyophilization. Subsequent to this, the section encompassing the epidermal layer was excised to harvest the dermal matrix. The final product, a dermal matrix measuring 10 mm × 10 mm × 2 mm, was precisely trimmed to the desired dimensions, preserved at -80 °C, and sterilized with phosphate-buffered saline (PBS) prior to application.

### Decellularization process

The dermal matrix sample was subjected to a freeze-thaw cycle to lyse cell membranes, initially frozen at -80 °C, subsequently thawed at 37 °C for 10 min, and this cycle was repeated 10 times. Following this, the sample was immersed in 1% Triton-X 100 and agitated at 100 rpm for 12 h, then rinsed with deionized water (DDH_2_O) for 1 h. The process continued with the sample being immersed in 1% sodium dodecyl sulfate (SDS) and stirred at 100 rpm for 6 h. To eliminate any residual reagents, the sample was finally cleaned with NaOH and HCl, stirring at 100 rpm for 24 h.

### Histological analysis

The Natural dermal matrix and acellular dermal matrix specimens were fixed in 4% paraformaldehyde for 24 h and subsequently embedded in paraffin wax. The wax-embedded blocks were then sectioned to a thickness of 5 μm using a microtome (CM1950, Leica, Germany). To assess the efficacy of the decellularization process, sections were stained with Hematoxylin and Eosin (H&E). DAPI staining was employed to confirm the absence of nuclei, and the DNA content was quantitatively evaluated using a DNA extraction kit. Given that Hydroxyproline (HYP) represents a consistently proportionate component of collagen, the collagen content was determined utilizing HYP assay kits.

### Preparation and characterization of silver nanoparticles (AgNPs)

First, prepare 30 mL of sodium borohydride solution (10 mM, 98% purity) and place it in an ice bath for subsequent use. Then, take 100µL of silver nitrate standard solution (0.5 M), pre-diluted to 10mL, and thoroughly mix it with 10mL of gallic acid (5mM). This mixture is then added dropwise to the sodium borohydride solution, maintaining stirring at 100 rpm for 5 h, with the entire procedure conducted in an ice bath. Upon completion of the reaction, the resultant gallic acid-modified silver nanoparticle solution is purified using a low-temperature high-speed centrifuge (15,000 rpm, 15 min).

For analysis, dilute the obtained silver nanoparticles and deposit 5µL drops onto a copper mesh; the size distribution is then measured via Transmission Electron Microscopy (TEM). To assess the stability of the silver nanoparticles over time, conduct separate experiments to monitor changes in UV absorbance at various intervals. Additionally, evaluate the UV absorbance fluctuations of the silver nanoparticles across different pH levels to gauge their stability under varying acidic or basic conditions.

### Ag@ADMH Preparation

The acellular dermal matrix was pulverized into powder, digested with 1% pepsin, and acidified with diluted hydrochloric acid to achieve a pH of approximately 2, ensuring complete digestion into a homogenate. To this homogenate, pre-cooled 40× phosphate-buffered saline (PBS) was added, followed by thorough mixing. Subsequently, pre-cooled NaOH solution (10mM) was incorporated into the mixture, with the pH adjusted to 7.2. This entire procedure was performed in an ice bath under sterile conditions. At this stage, the mixture attains a fluid dynamic state, referred to as “pre-hydrogel “. AgNPs- acellular dermal matrix hydrogel, designated as Ag@ADMH, is synthesized by integrating a concentrated solution of AgNPs into the prepared pre-hydrogel and stirring meticulously. Similarly, the acellular dermal matrix hydrogel (ADMH) is produced by adding an equivalent volume of deionized water (DDH_2_O) to the pre-hydrogel and ensuring thorough mixing. Upon standing at room temperature for a certain duration, the pre-hydrogel transitions into a hydrogel state, suitable for application in wound filling.

### Characterization of Ag@ADMH

To examine the spatial configuration of ADMH and Ag@ADMH, the critical point drying (CPD) method was employed for sample preparation, allowing for preservation of the samples’ native structure without the introduction of artifacts commonly associated with air drying. A select portion of the samples was segmented for observational analysis. The elemental composition and distribution within the samples were investigated, utilizing energy dispersive spectroscopy (EDS) for elemental analysis. Additionally, the alterations in functional groups and chemical states of the samples were explored through infrared spectroscopy (IR) and X-ray photoelectron spectroscopy (XPS), providing insight into the molecular interactions and surface chemistry of the hydrogels.

### Rheological analysis

The stability of ADMH and Ag@ADMH was analyzed at an angular frequency of 0.1 rad/s using a rotational rheometer, providing insights into the viscoelastic properties of these hydrogels. To assess the self-healing capabilities of ADMH and Ag@ADMH, macroscopic observation of incision and recombination was conducted. The criterion for successful healing was established as the ability of the hydrogel to maintain integrity when lifted at one end with tweezers, without separation under the influence of gravity, indicating robust self-healing properties. For quantitative analysis of the self-healing mechanism, the rheometer was employed at a microscopic level. Samples of ADMH and Ag@ADMH were positioned between plates with a diameter of 20 mm and a gap of 1.0 mm. A strain scan test was conducted, escalating the strain from 0.01 to 50% at a constant frequency of 1 Hz to evaluate the materials’ response to deformation and their ability to recover. This was followed by alternate strain scans conducted at 60-second intervals, maintaining a constant frequency of 1 Hz, all executed at room temperature. This methodology elucidates the hydrogels’ mechanical durability and their inherent self-repairing functionality.

The gelation time of ADMH and Ag@ADMH was analyzed using a time scan method, with a fixed frequency of 1 Hz and a strain of 20%, conducted at 37 °C.

### Biocompatibility of Ag@ADMH

To assess the cytotoxicity of ADMH and Ag@ADMH, the Cell Counting Kit-8 (CCK-8) assay was utilized. The samples were sterilized through irradiation and subsequently immersed in DMEM (hydrogel to medium ratio of 1:5) at 37 °C for 48 h. The leachate was then collected for analysis. Mouse fibroblasts (NIH 3T3) were seeded into 96-well plates at a density of 5 × 10^3 cells/well and cultured at 37 °C for 24 h. Following this, the supernatant was discarded, 100µL of the gradient leachate was added, and the medium was renewed daily (*n* = 3). Absorbance was measured at 450 nm after adding 10% CCK-8 reagent and incubating for 1 h at 37 °C.

For further analysis, a 100µL suspension of NIH3T3 cells was seeded in a 24-well plate at a density of 1 × 10^3 cells/well, with the hydrogel leachate being changed daily. The cells were maintained in a humidified atmosphere with 5% CO_2_ at 37 °C. At specific time points, cells were stained with calcein AM (2µM) and propidium iodide (PI) (4.5µM) and examined under a fluorescence microscope.

Hemocompatibility was evaluated by taking 10mL of blood from healthy rats, mixing it with an equal volume of normal saline, and centrifuging at 2000 rpm for 5 min. The supernatant was discarded, and the process was repeated three times to obtain washed red blood cells. These cells were prepared at a 2%(v/v) concentration with normal saline. The hydrogels were soaked in normal saline for 24 h (hydrogel to saline ratio of 1:5) to collect the leachate. Samples were divided into four groups: normal saline (negative control), DDH_2_O (positive control), ADMH, and Ag@ADMH. To each 1mL group, 0.5mL of the diluted washed red blood cells was added. After mixing and incubating at 37 °C for 3 h, the samples were centrifuged at 2000 rpm for 5 min. 200µL of the supernatant was then sampled, and absorbance was measured at 450 nm. The final optical density (OD) value was determined by subtracting the OD value of the blank from that of each sample.

To evaluate cell migration within the hydrogels, NIH3T3 cells were seeded at a density of 1 × 10^6 cells/mL on a 6-well plate. After 24 h of culture, a scratch was made in the confluent cell layer using the tip of a plastic straw, under the guidance of a ruler. Cell migration was observed under an optical microscope after the predetermined culture period, and mobility was calculated, with cells cultured in the absence of the hydrogel serving as the control.

### Ag@ADMH degradation in vitro

To assess the degradation of ADMH and Ag@ADMH under various conditions, 500 µg of each hydrogel were allocated into separate centrifuge tubes. Periodic inspections were conducted to monitor for any fluid secretion or complete disintegration of the hydrogels. Post-degradation, the hydrogel components were extracted, and the residual weight was quantified to evaluate the extent of degradation.

For a more detailed investigation, 500 µg of both ADMH and Ag@ADMH were placed in centrifuge tubes containing 1mL of PBS solution and 1mL of trypsin solution (2.5 mg/mL), and immersed for 8 days. The soaking solution was replaced daily with fresh solution, and the remaining weight of the hydrogels was calculated after each replacement. This entire procedure was conducted at 37 °C, enabling the simulation of conditions akin to the human body environment, thereby providing insights into the biodegradability and stability of the hydrogels in a biological context.

### AgNPs releasing performance of Ag@ADMH

To examine the impact of temperature on the release kinetics of silver nanoparticles from Ag@ADMH, 500 µg samples were distributed into centrifuge tubes, each containing 1mL of PBS solution. These samples were then incubated at three distinct temperatures: 25℃, 37℃, and 42℃, to represent a range from room temperature to febrile conditions. The release of silver nanoparticles into the PBS solution was monitored at various time intervals, enabling the evaluation of how temperature variations influence the diffusion rate and release mechanism of silver nanoparticles from the hydrogel matrix. This approach allows for a comprehensive understanding of the temperature-dependent release dynamics of AgNPs, which is crucial for optimizing therapeutic efficacy and safety in clinical applications.

### Antibacterial ability

To assess the antibacterial efficacy of Ag@ADMH hydrogel, an in vitro study was conducted against methicillin-resistant Staphylococcus aureus (MRSA). MRSA at its logarithmic growth phase was prepared as a bacterial suspension with a concentration of 1 × 10^6 CFU/mL. This suspension was then co-cultured with Ag@ADMH hydrogel for 24 h at 37℃. The optical density (OD) values were measured at 600 nm at various intervals to monitor bacterial growth or inhibition. Following this, the spread plate method (SFM) was utilized to quantify the colonies formed, facilitating the calculation of the hydrogel’s antibacterial efficiency. This procedure was replicated three times to ensure the reliability of the results. Furthermore, to gain insights into the interaction between the bacteria and the hydrogel, samples from the co-culture were examined under Transmission Electron Microscopy (TEM). This allowed for the detailed visualization of any structural changes or damage to the MRSA cells induced by the silver nanoparticles embedded within the hydrogel, providing a microscopic understanding of the antibacterial mechanism at play.

### Antioxidant capacity

The antioxidant capacity of Ag@ADMH hydrogel was evaluated through its DPPH (2,2-diphenyl-1-picrylhydrazyl) free radical scavenging activity, which is a measure of its ability to inhibit DPPH free radicals. Samples of ADMH, Ag@ADMH, and ascorbic acid (serving as a positive control) at various concentrations were prepared and reacted with 1mL of DPPH solution (0.2mM) in a light-protected environment for 30 min. Subsequently, the absorbance of each reaction mixture was measured at a wavelength of 517 nm.

The inhibition rate of DPPH free radicals by the samples was calculated using the formula:

$$\:\text{Inhibition\:rate}\left(\text{\%}\right)=\left(\frac{{A}_{\text{control}}-{A}_{\text{sample}}}{{A}_{\text{control}}}\right)\times\:100$$

where A_control_ is the absorbance of the DPPH solution without any sample added (control), and A_sample_ is the absorbance of the DPPH solution after reaction with the sample. This calculation provides a quantitative measure of the sample’s antioxidant activity, with higher inhibition rates indicating stronger radical scavenging abilities.

### In vitro anti-inflammatory ability

To evaluate the immunomodulatory effects of the scaffold on RAW264.7 macrophages, cells were seeded onto the specimens at a density of 1 × 10^5 cells per well. Following a 24-hour incubation period, RNA was extracted using a commercial kit, and reverse transcription was performed according to the kit’s protocol. The expression levels of the housekeeping gene GAPDH were used to normalize the expression of target genes, facilitating accurate comparisons.

For the detection of M1 (iNOS) and M2 (CD206) markers in macrophages, immunofluorescence was employed. RAW264.7 cells were stimulated with 200ng/mL lipopolysaccharide (LPS) for 18–24 h to induce an M1 phenotype and then co-cultured with the scaffolds at 1 × 10^5 cells/well for an additional day. Post-culture, macrophages were transferred to a new plate for reattachment. Cells were then fixed with 4% paraformaldehyde/PBS, permeabilized with Triton X-100, and blocked with 5% normal goat serum for 1 h. Primary antibodies (ab210823 for iNOS and ab64693 for CD206) were applied overnight at 4℃, followed by incubation with secondary antibodies (ab6785 and ab6939) for 1 h. DAPI staining for 10 min was performed before laser confocal microscopy analysis.

Additionally, the surface expression of M1 (CD11c) and M2 (CD206) markers on macrophages was quantified using flow cytometry, as previously described [28]. RAW264.7 cells were induced with 200ng/mL LPS and cultured for 1 day. Inflammation-induced cells were then co-cultured with the hydrogel, and after 24 h, cells were collected, centrifuged at 300×g for 5 min, washed twice with PBS, and blocked with 1% bovine serum albumin for 30 min. Cells were stained with PE-conjugated CD11c and APC-conjugated CD206 antibodies. Flow cytometry (Agilent Technologies, Nanocyte 2060R) was utilized to analyze the labeled cells, providing insights into the polarization dynamics of macrophages in response to the scaffold’s immunomodulatory properties.

### Mouse subcutaneous balloon model

To investigate the immunomodulatory effects of the hydrogel, a balloon model was established in nine C57BL/6 mice, aged 8 weeks. These mice were divided into three groups, with three mice per group designated for HE (Hematoxylin and Eosin) staining to assess tissue reactions. The mice were anesthetized via intraperitoneal injection of 1% pentobarbital sodium at a dosage of 50 mg/kg. To initiate the model, 3 mL of sterile air was injected into the subcutaneous tissue on the backs of the mice. Four days later, this procedure was repeated twice with the same volume of sterile air to ensure the formation of a sufficient space that simulates a balloon effect. One day following the second air injection, a surgical incision was made on the backs of the mice, and a pocket was carefully created under the skin using blunt dissection. Into this pocket, 100µL of sterilized hydrogel was injected. All procedures were carried out under strict aseptic conditions to prevent infection and ensure the integrity of the experiment. This model allows for the evaluation of the hydrogel’s capacity to modulate the immune response in a controlled, localized environment, providing valuable insights into its potential for clinical applications in wound healing and tissue engineering.

### Model of infective skin defect

In a study involving 3-month-old male Sprague-Dawley rats weighing 400–450 g, a full-thickness skin defect model was established across three groups. Prior to the surgical procedure, rats were anesthetized with ketamine (30.0 mg/kg). The surgical site on the back was prepared by removing hair and marking a 12 mm circle where the skin defect was then created using a tissue examination perforator. To simulate infection, 20µL of methicillin-resistant Staphylococcus aureus (MRSA) was applied to the wound for several hours. The control group received a Tegaderm wound dressing directly applied to the wound site. In contrast, the experimental groups were treated with injections of ADMH and Ag@ADMH hydrogel, respectively. The wound healing process was monitored over 7 and 14 days, with wound size measurements recorded at both intervals. For histological analysis, HE staining was conducted to assess epithelial regeneration and hair follicle count; Masson’s trichrome staining was used to evaluate collagen content; CD68 and Myeloperoxidase (MPO) immunohistochemistry were performed to analyze inflammatory response; and CD34 staining was utilized to investigate angiogenesis. This comprehensive approach allowed for a detailed evaluation of the hydrogels’ effects on wound healing, tissue regeneration, inflammation, and blood vessel formation in a clinically relevant model.

### Statistical analysis

Data are presented as mean ± standard deviation. Differences between two groups were analyzed using a two-tailed Student’s t-test, while differences among multiple groups were assessed with one-way ANOVA. A P-value less than 0.05 was considered statistically significant.

## Results and discussion

### Preparation of acellular dermal matrix and hydrogel

**Fig. 1:**
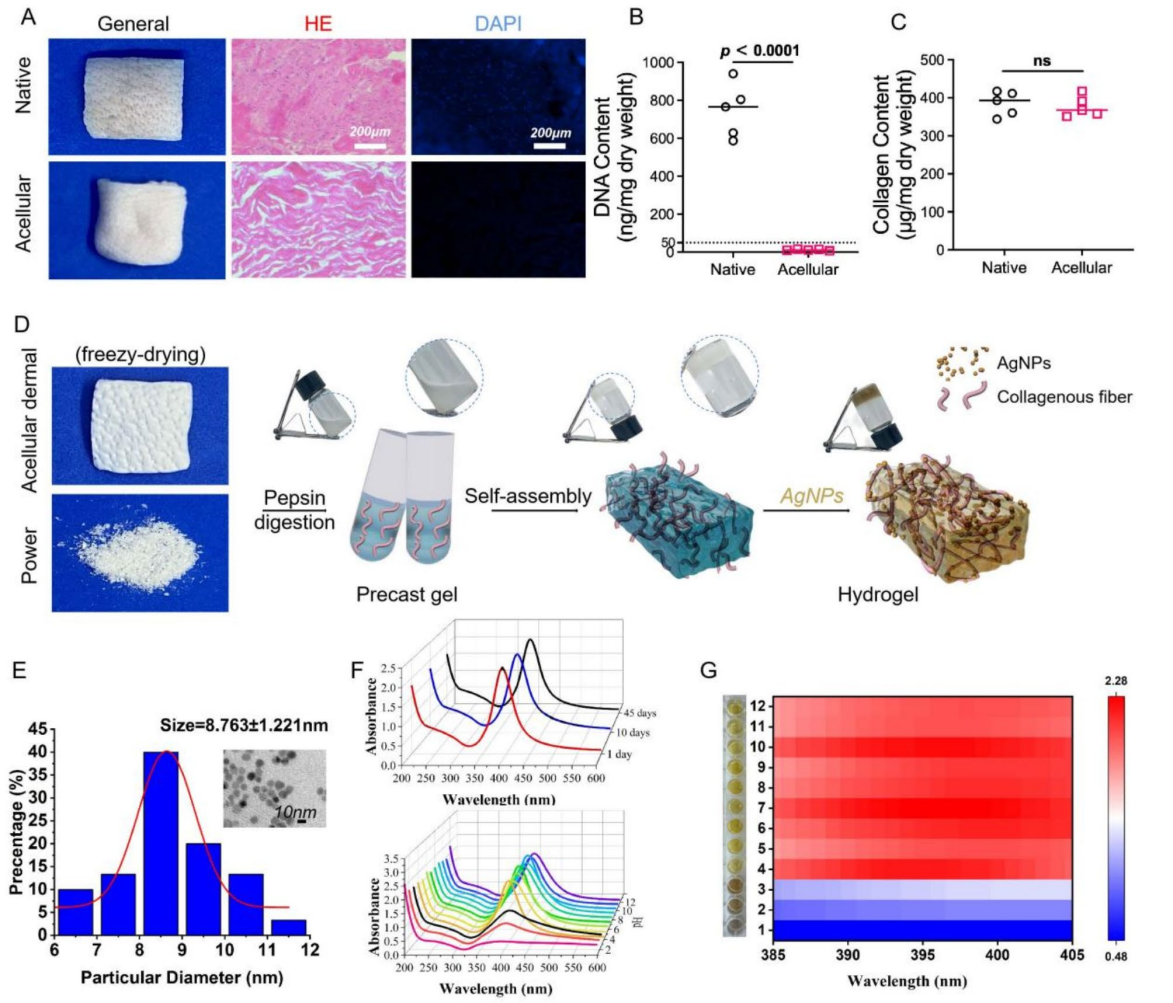
Characterization of the dermal matrix hydrogel. **A** Macroscopic image, H&E, and DAPI staining. **B** Analysis of DNA content. **C** Analysis of collagen content. **D** Preparation process of Ag@ADMH. **E-G** Particle size analysis of AgNPs, time stability, and pH stability

The dermal extracellular matrix (ECM) is a densely fibrotic and porous three-dimensional network that facilitates cell growth and directs tissue regeneration. The acellular dermal ECM, characterized by fibers of nanoscale and micrometer-scale dimensions, offers a natural microenvironment that provides structural and mechanical support as well as biochemical cues essential for cell adhesion, proliferation, and differentiation. Moreover, the dermal ECM exhibits a high affinity for various cells, playing a vital role in mediating the wound healing process. Initial H&E and DAPI staining of the acellular dermal ECM post-treatment with Triton-X100 and sodium lauryl sulfate (SDS) through a freeze-thaw cycle indicated a thorough removal of cellular elements (Fig. 1A). Subsequent quantitative DNA analysis revealed a significant reduction in DNA content compared to natural tissues (745.2 ± 142 vs. 11.97 ± 3.78, *p* < 0.0001) (Fig. 1B). Despite the decellularization process, collagen—the main structural component of the dermal matrix—showed no significant alteration in quantity (385.1 ± 32.04 vs. 377.2 ± 27.09, *p* > 0.05) (Fig. 1C), suggesting the preservation of crucial dermal ECM components. Following this, the acellular dermis ECM was freeze-dried under vacuum, pulverized into a fine powder, and the collagen decomposed into fibers using pepsin, subsequently neutralized with PBS and sodium hydroxide. These collagen fibers were reassembled into larger fibrous networks, all conducted on ice, resulting in a pre-hydrogel state with fluidity that transitions to a hydrogel upon heating to 37℃ for several minutes. Plant polyphenol AgNPs, synthesized as previously described [22], were incorporated into the pre-hydrogel, altering the hydrogel’s color to light yellow and ensuring thorough mixing via ultrasound for 1 min, thus forming the AgNPs -embedded dermal matrix hydrogel (Ag@ADMH). This entire process was conducted on ice (Fig. 1D). TEM analysis of the AgNPs demonstrated uniform dispersion with an average size of 8.763 ± 1.221 nm (Fig. 1E). To ascertain the stability of the AgNPs, their properties were analyzed and at different pH levels. The maximum absorption peak remained relatively unchanged after a month, while significant alterations in appearance and absorbance were observed at pH < 4 (Fig. 1F). A heatmap further illustrated a sharp decrease in absorbance at pH = 3 (Fig. 1G), which may due to the protonation of gallic acid molecules under strong acidic conditions, leading to nanoparticle agglomeration.

Considering the skin’s naturally weakly acidic surface and the alkaline conditions typically found in chronic or infected wounds, the engineered AgNPs demonstrate stability across both physiological and pathological conditions [29]. This adaptability to the changing pH of healing wounds underscores the potential of Ag@ADMH hydrogels in enhancing wound care, particularly for chronic or infected wounds, by leveraging the synergistic effects of ECM scaffolds and antibacterial silver nanoparticles.

### Characterization of silver nanoparticles integrated dermal matrix hydrogels (Ag@ADMH)

**Fig. 2:**
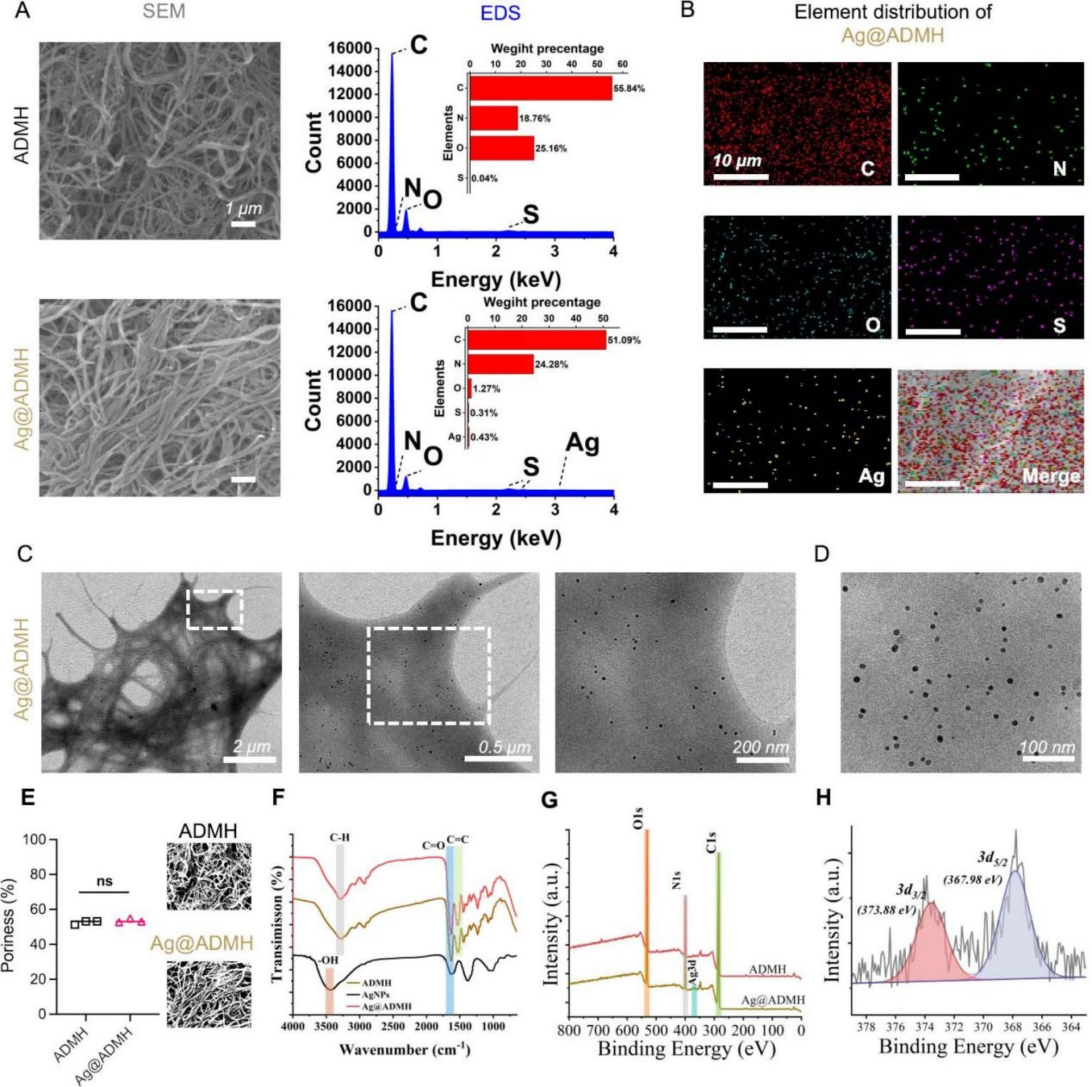
**A**,** B** SEM images of ADMH and Ag@ADMH, with EDS elemental analysis. **C** TEM image analysis of Ag@ADMH. **D** TEM results of AgNPs released from Ag@ADMH. **E** Pore size analysis of ADMH and Ag@ADMH. **F** FTIR spectra results of AgNPs, ADMH, and Ag@ADMH. **G**,** H** XPS results of ADMH and Ag@ADMH, including the 3d spectrum of Ag

The Scanning Electron Microscopy (SEM) examination elucidated the structural interplay between ADMH and Ag@ADMH. It was observed that the nanofiber network structure suggests that collagen can reassemble into collagen-like fibrous structures post-digestion by gastric protease (Fig. 2A), enhancing the affinity for silver nanoparticle attachment. Energy Dispersive Spectroscopy (EDS) analysis systematically investigated the elemental composition of the hydrogels, revealing the presence of 0.43% silver within Ag@ADMH, uniformly distributed albeit in minimal quantities (Fig. 2B). Transmission Electron Microscopy (TEM) further scrutinized Ag@ADMH’s microstructure (Fig. 2C), corroborating the fibrous network observed via SEM and identifying dispersed silver nanoparticles. To explicitly observe Ag@ADMH’s silver nanoparticle release behavior, an equal volume of Ag@ADMH and PBS was combined in a centrifuge tube and agitated. The resultant solution was analyzed under TEM to observe the uniformly distributed nanoparticles (Fig. 2D). Both SEM and TEM verified that Ag@ADMH preserves an intact fiber network structure. Porosity analysis conducted with ImageJ (Fig. 2E) demonstrated no significant variance in porosity following silver nanoparticle integration (ADMH vs. Ag@ADMH, 52.56 ± 1.119 vs. 53.57 ± 1.172, *p* > 0.05), indicating that nanoparticle adsorption does not affect the scaffold’s porosity. Fourier Transform Infrared Spectrometer (FTIR) analysis (Fig. 2F) further affirmed that AgNPs within Ag@ADMH are adsorbed rather than chemically bonded, with the FTIR spectrum of Ag@ADMH showing substantial overlap with that of AgNPs, including unique absorption peaks absent in ADMH, such as the benzene ring skeleton vibration peak near 1500 cm^− 1^. X-ray Photoelectron Spectroscopy (XPS) analysis (Fig. 2G) with carbon correction highlighted an Ag3d peak in Ag@ADMH, with the Ag fine spectrum (Fig. 2H) splitting the Ag3d orbit into 3d5/2 (367.98 eV) and 3d3/2 (373.88 eV) orbits. These findings indicate that the adsorption of AgNPs within Ag@ADMH does not compromise the hydrogel’s porosity while ensuring favorable nanoparticle dispersion.

**Fig. 3:**
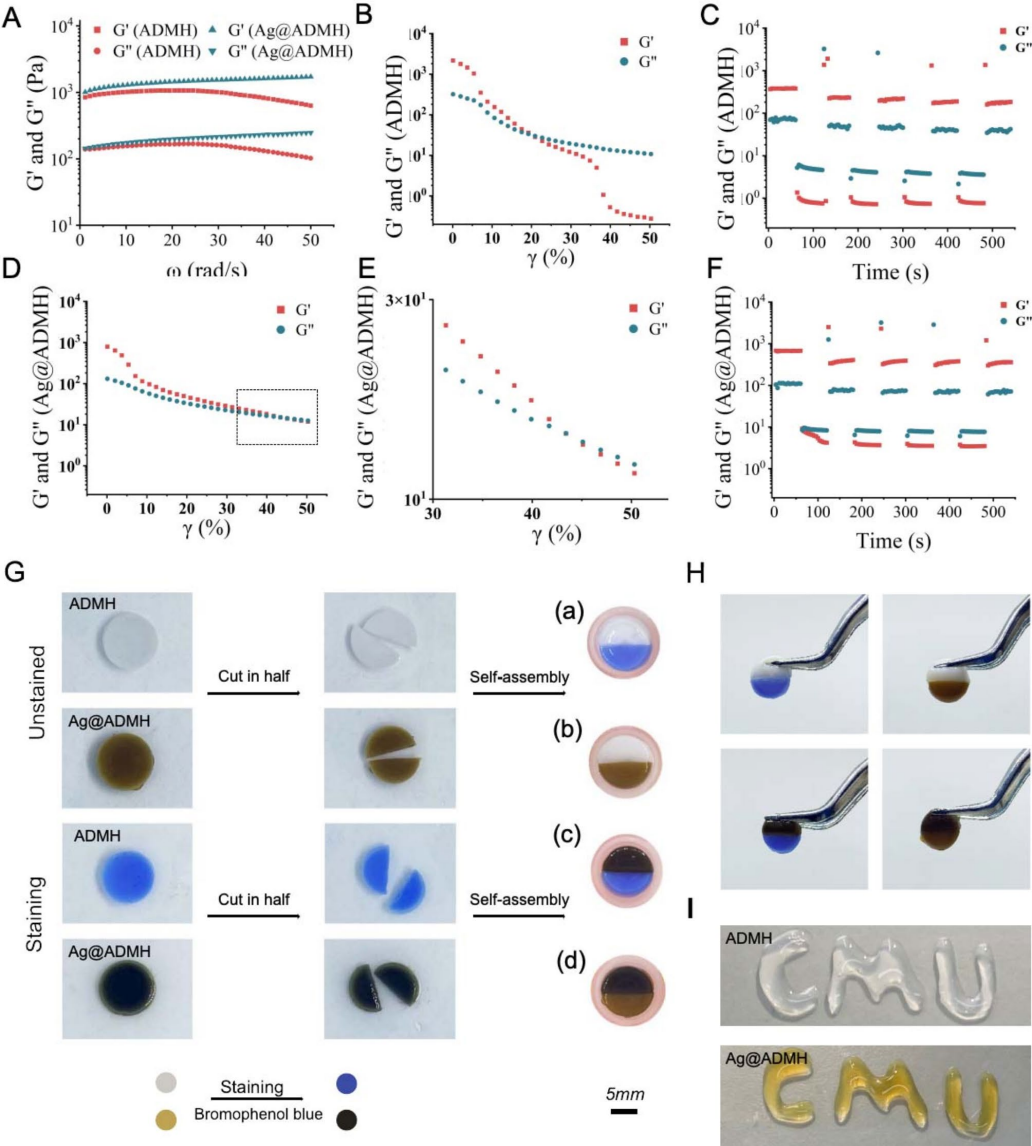
Rheological analysis and self-healing properties of the hydrogel. **A** Rheological analysis of ADMH and Ag@ADMH as a function of angular frequency at room temperature. **B** Rheological analysis of ADMH as a function of shear strain at room temperature. **C** Self-healing property of ADMH. **D** Rheological analysis of Ag@ADMH as a function of shear strain at room temperature, further illustrated in **E**. **F** Self-healing property of Ag@ADMH. **G** Macroscopic self-healing experiments of ADMH and Ag@ADMH using bromophenol blue dye for hydrogel staining. **H** Images of hydrogel healing after 30 min of cutting. **I** Optical images of the injectability of the hydrogel

The energy storage modulus (G’) and loss modulus (G”) of both hydrogels (ADMH and Ag@ADMH) were assessed via a rheometer’s scanning frequency range of 1 to 50 rad/s. In Fig. 3A, the G’ and G” of Ag@ADMH were found to be superior to those of ADMH, indicative of their hydrogel state. This enhancement is attributed to the gallic acid molecule’s pyrogallol group, which harbors three hydroxyl groups capable of forming numerous hydrogen bonds through intermolecular and intramolecular interactions. Given these extensive hydrogen bonding capabilities, Ag@ADMH is postulated to exhibit self-healing properties, enabling it to reconstitute its mechanical integrity post-destruction by external forces. Rheometric analysis revealed that for ADMH, G” surpasses G’ at around 20% strain (Fig. 3B), marking the point of hydrogel structural compromise. Using this threshold as a reference, alternating fixed strains of 0.01% and 50% were applied, demonstrating fluctuations in G’ values under varying strain conditions (Fig. 3C). This behavior indicated that ADMH undergoes damage at high strain and swiftly recuperates at low strain, showcasing reversible damage-repair dynamics and thus, self-healing capabilities. Similarly, Ag@ADMH was subjected to analysis (Fig. 3D), with findings indicating greater G’ and G” intersections compared to the ADMH group devoid of AgNPs. Closer inspection revealed a critical strain range between 40% and 50% (Fig. 3E), potentially due to increased hydrogen bonding facilitated by the gallic acid molecule. Adjusting the alternating strain to 0.01% and 60%, Ag@ADMH also displayed varying G’ values under these conditions, affirming its robust self-healing potential (Fig. 3F). A macroscopic self-healing test was conducted by bisecting both ADMH and Ag@ADMH hydrogels and rejoining them. To visually track the healing process, bromophenol blue was incorporated as a dye during preparation. Left at room temperature without external intervention (Fig. 3G), the hydrogels demonstrated cohesion within minutes, enabling the lifting of the gel pieces with tweezers, thus confirming their adhesion (Fig. 3H). As illustrated in Fig. 3I, Ag@ADMH showcases exceptional injectability and moldability, underscoring its suitability for filling irregular wounds and enhancing its practical applicability. These results collectively affirm Ag@ADMH’s effective self-healing properties, injectability, and utility in wound management.

**Fig. 4:**
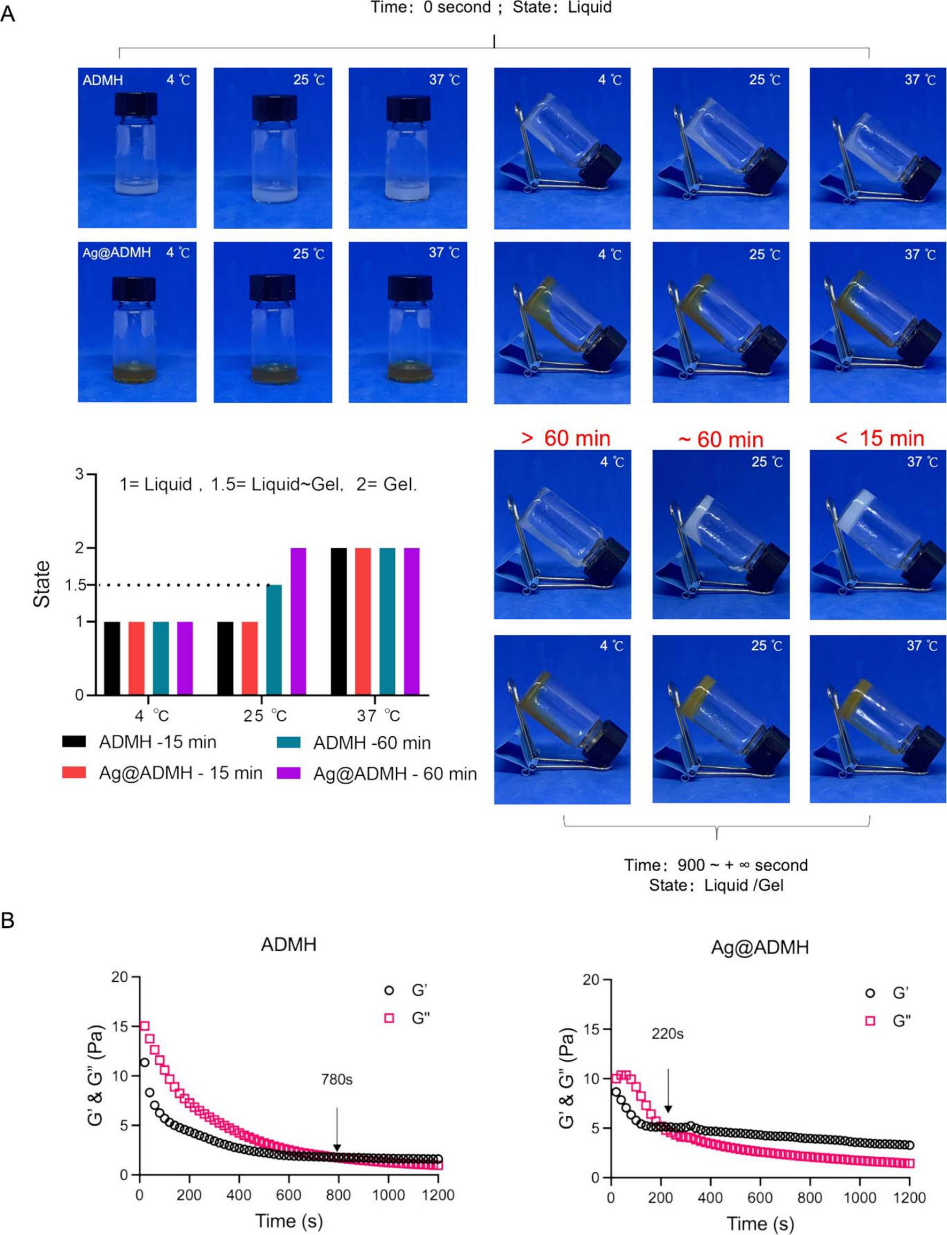
Temperature sensitivity analysis of the hydrogel. **A** Gelation time of ADMH and Ag@ADMH measured at 4 °C, 25 °C, and 37 °C. **B** Further analysis of gelation time of ADMH and Ag@ADMH at 37 °C using a rheometer

To assess the gelation time of synthesized ADMH and Ag@ADMH, hydrogel states were initially observed at 4 °C, 25 °C, and 37 °C. As depicted in Fig. 4A, all samples initially appeared in a liquid state. After 15 min of observation, the hydrogel in the 37 °C group first transitioned from a liquid to a hydrogel state. Subsequently, after extending the observation time to 60 min, the hydrogel in the 25 °C group showed that Ag@ADMH had largely formed into a hydrogel state, whereas ADMH exhibited partial fluidity. Concurrently, the hydrogel in the 4 °C group remained in a liquid state with observable fluidity. Further analysis was conducted on the 37 °C group using a rheometer for time scan testing, with the experimental temperature held constant at 37 °C. As shown in Fig. 4B, the gelation time of ADMH at 37 °C was 780 s, while that of Ag@ADMH was approximately 220 s. The observed difference may be attributed to the introduction of GA and the interplay between hydrogen bonds and π-π bonds, which likely accelerated hydrogel formation [30, 31].

### Biocompatibility of Ag@ADMH

**Fig. 5:**
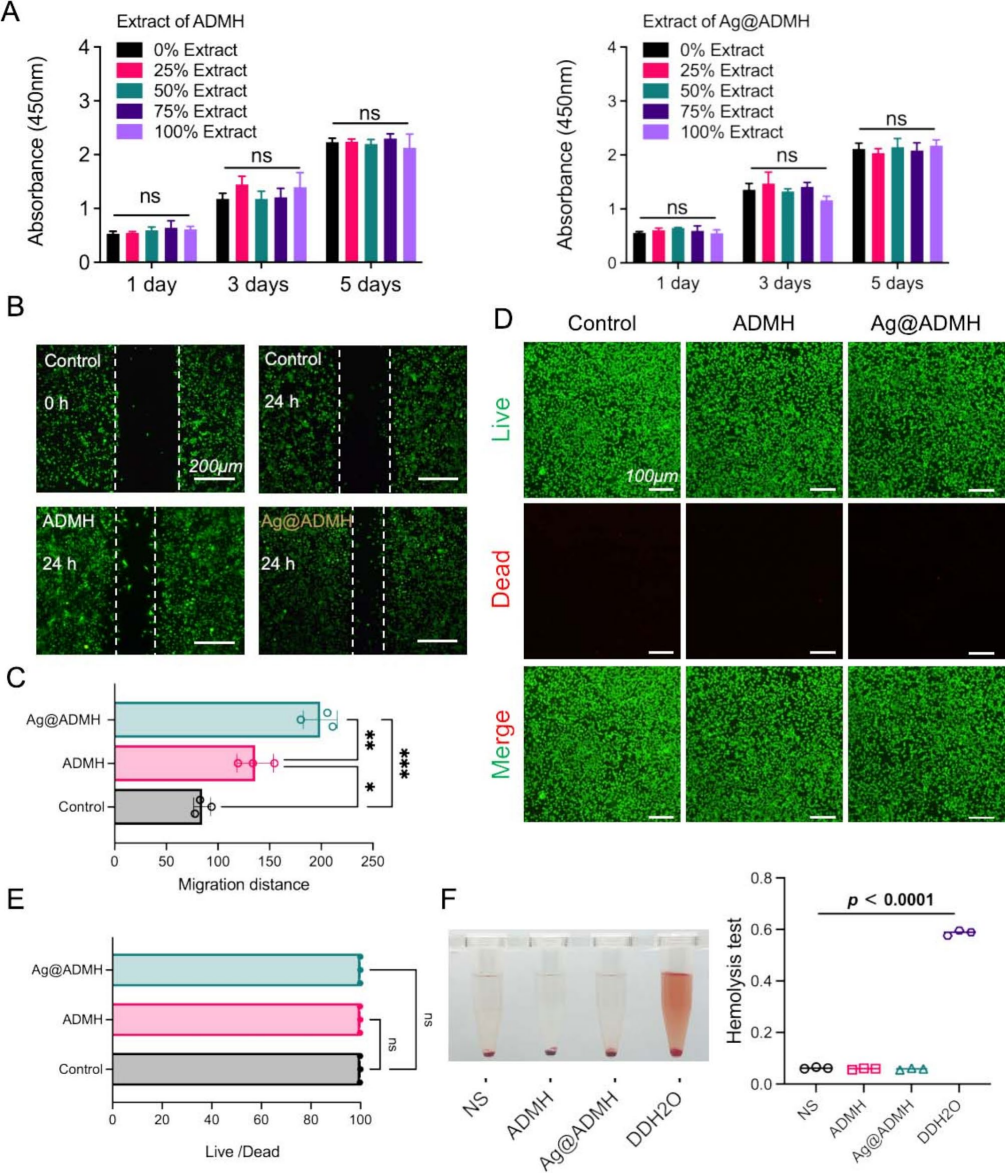
Biocompatibility. **A** Toxicity analysis of NIH3T3 cells cultured in soaking solutions of ADMH and Ag@ADMH at different concentrations for 1, 3, and 5 days using the CCK-8 assay. **B**,** C** Migration and analysis of NIH3T3 cells in the hydrogel environment. **D**,** E** Fluorescence imaging and analysis of NIH3T3 cells in the hydrogel environment. Live cells stained with calcein-AM (green), and dead cells stained with propidium iodide (red). **F** Hemolysis assay, with physiological saline (ns) and distilled water serving as negative and positive controls, respectively

The cytotoxicity of ADMH and Ag@ADMH was assessed through a CCK-8 assay (Fig. 5A). Throughout the culturing period, no notable reduction in cell metabolic activity was observed in the control group (NIH3T3 co-cultured with DMEM) compared to the experimental groups (NIH3T3 combined with 25%, 50%, 75%, and 100% DMEM infusion of ADMH/Ag@ADMH, respectively). Subsequently, the migration of NIH3T3 cells cultured in the Ag@ADMH soak solution was evaluated, as depicted in Fig. 5B, C. Relative to cells seeded in pore plates, Ag@ADMH markedly facilitated the migration of NIH3T3 cells. Cell viability in the presence of ADMH and Ag@ADMH hydrogels was further scrutinized using calcein-propyl iodide staining (Fig. 5D, E). Following a 48-hour co-culture with the hydrogels, calcein staining indicated no significant disparity in the quantity of live cells between ADMH and Ag@ADMH and the live/dead cells on the surface of the blank group, signifying excellent cell compatibility. A hemolysis test (Fig. 5F) was conducted to evaluate blood compatibility, with normal saline and distilled water serving as controls. Similar to the normal saline group, both the ADMH and Ag@ADMH groups showed that all erythrocytes settled, the supernatant remained colorless and transparent, and no hemolysis was observed. In contrast, hemolysis was evident in the distilled water group. Quantitative analysis of the hemolysis test revealed no significant difference in the OD value of Ag@ADMH compared with the normal saline (NS) group (*P* > 0.05, *n* = 3), but a notable difference when compared with the distilled water (H_2_O) group (*P* < 0.0001). These findings underscore that Ag@ADMH not only exhibits excellent cell compatibility, but also promotes cell proliferation and migration in vitro, thereby supporting the healing of damaged tissues.

### Degradation and the silver nanoparticles release capacity of Ag@ADMH

**Fig. 6:**
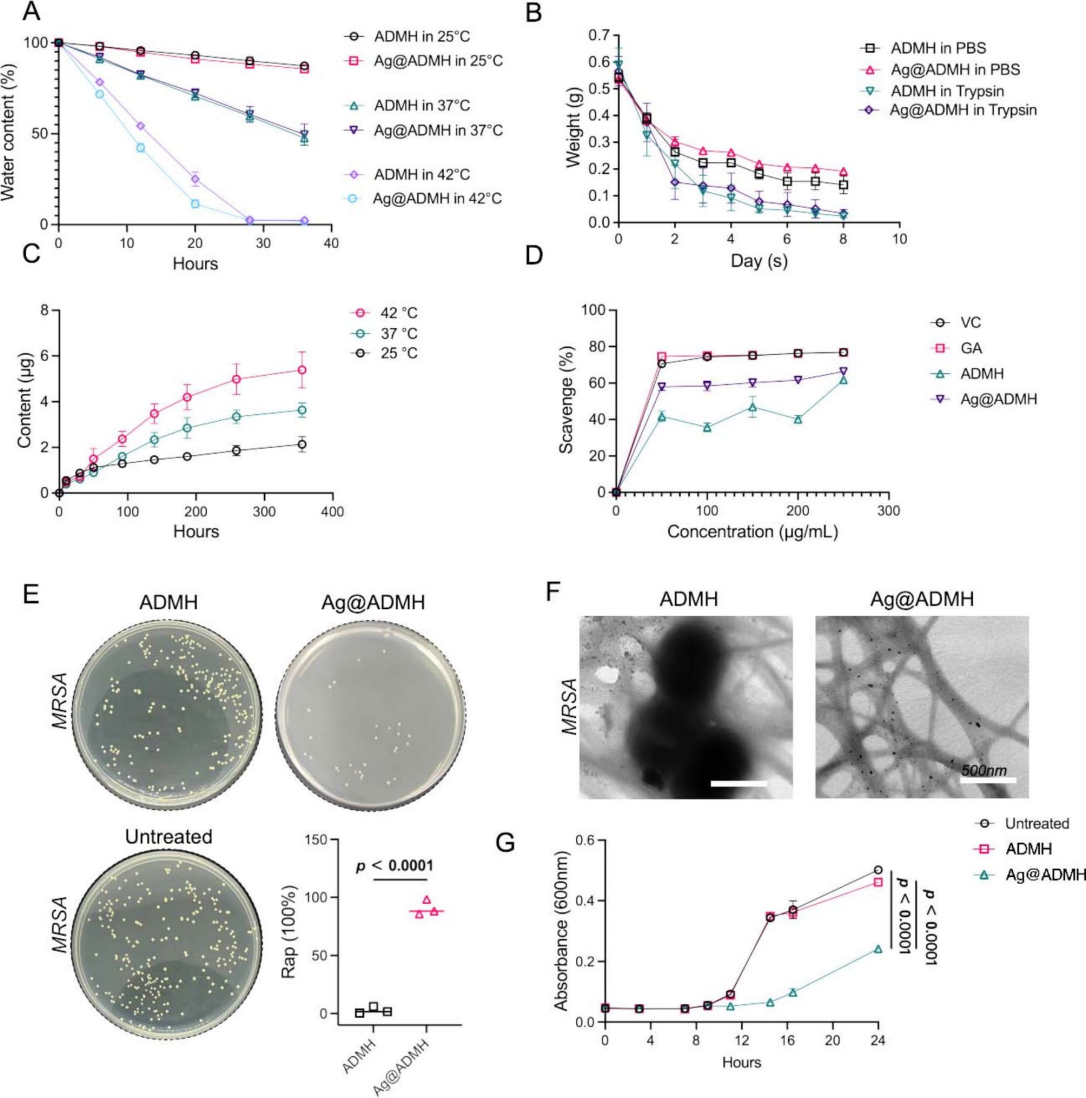
**A**,** B** Analysis of degradation capability of ADMH and Ag@ADMH at different temperatures and in different environments. **C** Release capacity of AgNPs from Ag@ADMH. **D** Oxygen free radical scavenging capability of ADMH and Ag@ADMH. **E** Antimicrobial results and analysis of ADMH and Ag@ADMH against MRSA. **F** Attachment of MRSA to ADMH and Ag@ADMH observed under TEM. **G** Variation in bacterial concentration of ADMH and Ag@ADMH over different time intervals

When tissue damage and microbial infections provoke local inflammatory reactions, they often lead to increases in local temperature [32]. To assess the in vitro biodegradability of ADMH and Ag@ADMH, experiments were conducted at temperatures of 25 °C, 37 °C, and 42 °C. As illustrated in Fig. 6A, the degradation rates of both ADMH and Ag@ADMH hydrogels escalated with rising temperatures. The breakdown of the hydrogel at 25 °C was prolonged, while a marked acceleration in degradation was observed with temperature elevation. Specifically, at 42 °C, the hydrogel underwent near-complete degradation within approximately one day. Conversely, at 37 °C, reaching a 50% degradation level required over 36 h. This temperature-dependent degradation behavior underscores the adaptability of ADMH and Ag@ADMH hydrogels to varying physiological conditions, potentially enabling tailored degradation rates suitable for different phases of the wound healing process.

Trypsin and PBS were utilized to further investigate the degradability of ADMH and Ag@ADMH (Fig. 6B). Figure 6B reveals that the degradation rate of ADMH in PBS was marginally faster than that of Ag@ADMH; by day 4, the degradation rates were 58.94 ± 2.758% for ADMH and 51.1 ± 2.585% for Ag@ADMH, *p* < 0.05, indicating a quicker degradation of ADMH possibly due to the fewer hydrogen bonds within its structure. By day 8, the degradation rates adjusted to 74.01 ± 6.198% for ADMH and 64.25 ± 0.83% for Ag@ADMH, *p* > 0.05, suggesting that the gradual release of silver nanoparticles as degradation progressed diminished the disparity caused by hydrogen bonding. Conversely, in the trypsin degradation system, the degradation rates by day 4 were 84.47 ± 8.061% for ADMH and 77.25 ± 9.97% for Ag@ADMH, *p* > 0.05; by day 8, these rates increased to 96.04 ± 0.98% and 94.01 ± 2.7%, respectively, *p* > 0.05. There was no significant difference in the hydrogel degradation rates between the two groups over different periods, but both were higher than those observed with PBS treatment. This could be attributed to the dermal ECM being broken down into various collagen fibers during hydrogel preparation, with the ADMH hydrogel containing trypsin-degradable protein components. These findings imply that locally released proteases during tissue damage may degrade other hydrogel protein components, facilitating the release of silver nanoparticles. The release rate of silver nanoparticles from Ag@ADMH at 25 °C, 37 °C, and 42 °C was also measured (Fig. 6C), revealing an increase in release corresponding with rising temperatures, indicative of temperature sensitivity. This suggests that in response to local injury or infection—where an increase in local temperature occurs—the release of silver nanoparticles is enhanced, thereby exerting an anti-inflammatory effect and promoting local tissue repair.

In response to the acidic microenvironment observed during wound infections [14], we investigated the degradation behavior of ADMH and Ag@ADMH, as well as the release behavior of AgNPs under pH conditions of 5 and 7.4. Over a continuous 4-day observation period, we found no significant differences in the degradation of ADMH and Ag@ADMH between the two pH levels (Fig. S1). This suggests that the hydrogel exhibits minimal pH sensitivity in terms of degradation, allowing it to remain stable and ensure a controlled, sustained release of the drug across different pH environments typically present in wounds. However, the release of AgNPs exhibited marked differences (Fig. S2). The amount of AgNPs released at pH = 5 was significantly higher than that of condition with pH = 7.4. This discrepancy is likely due to the presence of gallic acid on the surface of the AgNPs, acting as a capping agent. In acidic conditions, gallic acid tends to undergo protonation [33], which may facilitate a greater release of AgNPs.

The above results indicate that the release of AgNPs from Ag@ADMH is sensitive to both temperature and pH, aligning with the microenvironment of infected wounds, where local temperature rises and pH becomes more acidic. This characteristic endows Ag@ADMH with significant potential for use in treating infected wounds, enabling targeted drug release at the site of infection, thereby accelerating the healing process and enhancing therapeutic outcomes.

### Antioxidant capacity of Ag@ADMH

The 2,2-diphenyl-1-picrylhydrazyl (DPPH) assay, a marker for free radical scavenging activity, evaluates the ability of antioxidants to donate H atoms to stabilize and neutralize free radicals. This method was employed to assess the antioxidant capabilities of ADMH and Ag@ADMH. Figure 6D presents the free radical scavenging activities of ascorbic acid, gallic acid, ADMH, and Ag@ADMH at varying concentrations. The findings indicated that ascorbic acid, gallic acid, and Ag@ADMH exhibited high antioxidant activities at concentrations around 50 µg/mL. Notably, ascorbic acid and gallic acid consistently outperformed Ag@ADMH in antioxidant potential, with their anti-inflammatory activity enhancing proportionally to the increase in their concentrations. Conversely, ADMH demonstrated a comparatively weaker antioxidant capacity, underscoring the significant role that the incorporation of silver nanoparticles and gallic acid plays in bolstering the antioxidant and anti-inflammatory properties of Ag@ADMH.

### Antibacterial activity of Ag@ADMH

Drug-resistant bacterial infections pose a significant challenge to effective skin regeneration. In this context, the antibacterial activity of ADMH and Ag@ADMH against methicillin-resistant Staphylococcus aureus (MRSA) was evaluated. Bacterial suspensions (1 × 10^6 CFU/mL) were co-cultured with ADMH and Ag@ADMH for 24 h. Figure 6E illustrates the antibacterial efficacy of both ADMH and Ag@ADMH as quantified by the Spread Plate Method (SPM). Notably, there was a discernible difference in the bacterial colony counts between the plates corresponding to ADMH and Ag@ADMH, with bactericidal rates of 2.710 ± 2.996% and 90.790 ± 6.684%, respectively. Further analysis through Transmission Electron Microscopy (TEM) (Fig. 6F) revealed that numerous MRSA cells were adhered to ADMH, whereas Ag@ADMH samples showed no such adherence, only presenting scattered silver nanoparticles. This observation suggests a significant antibacterial property inherent to Ag@ADMH. The growth curves of MRSA cultured with ADMH and Ag@ADMH were also delineated. As depicted in Fig. 6G, ADMH exhibited a limited inhibitory effect on MRSA growth, in stark contrast to Ag@ADMH, which significantly curtailed MRSA proliferation. These results align with the findings from the SPM analysis, affirming that Ag@ADMH possesses a robust inhibitory effect on drug-resistant bacteria. This efficacy not only underscores the potential of Ag@ADMH in combating bacterial infections but also highlights its utility in facilitating the repair of infected wounds, thereby addressing a critical barrier in skin regeneration.

### Macrophage polarization

Evidence from prior research indicates that macrophages differentiated into the M1 subtype are known for secreting pro-inflammatory mediators, which are crucial during the initial response to infection or injury by activating immune responses. On the contrary, M2 macrophages are characterized by their production of anti-inflammatory cytokines and therapeutic growth factors, contributing significantly to the establishment of an anti-inflammatory microenvironment [34]. This dichotomy underscores the pivotal role of macrophages as primary responders in inflammation, playing a central role in both pathogen eradication and tissue repair processes [35]. Their dynamic functionality highlights the importance of understanding macrophage behavior in the context of wound healing and regenerative medicine, pointing towards their potential as targets for therapeutic interventions aimed at enhancing tissue repair and combating infections.

**Fig. 7:**
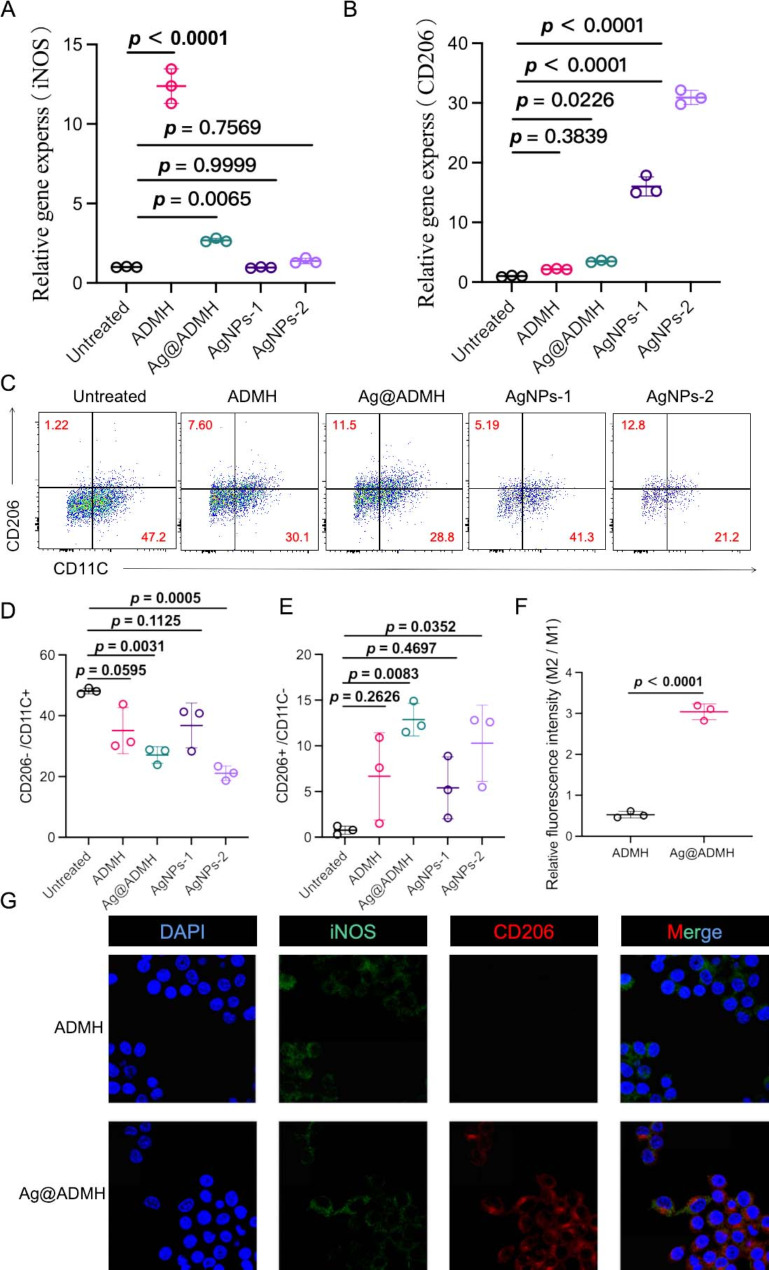
Macrophage chemotaxis. **A**,** B** Expression analysis of iNOS (M1) and CD206 (M2) by RT-qPCR. **C-E** Flow cytometry analysis of CD11C (M1) and CD206 (M2) expression levels, with quantitative analysis. **F**,** G** Fluorescence staining analysis of iNOS (M1) and CD206 (M2) expression in macrophages co-cultured with ADMH and Ag@ADMH, with analysis of M2/M1 ratio

Quantitative Reverse Transcription Polymerase Chain Reaction (qRT-PCR) was employed to assess the gene expression levels of macrophage-related markers M1 (iNOS) and M2 (CD206), crucial for evaluating the immunomodulatory effects of Ag@ADMH. In a lipopolysaccharide (LPS)-induced inflammation model (200 ng/mL), cells were cultured with ADMH and Ag@ADMH for one day. qPCR analysis revealed a significant elevation in iNOS expression within the ADMH group (Fig. 7A, B), indicating a pronounced pro-inflammatory response. This finding aligns with prior research, suggesting potential limitations in the direct application of dermal ECM due to its pro-inflammatory tendencies. Conversely, the Ag@ADMH group exhibited an increase in iNOS expression albeit at a reduced level compared to the ADMH group, likely mitigated by the incorporation of silver nanoparticles. The presence of phenolic hydroxyl groups in these nanoparticles may contribute to inflammation inhibition and address the issue of pro-inflammatory expression associated with dermal ECM. Further investigation into the inflammatory expression profiles at lower concentrations of silver nanoparticles (5 µg/mL and 10 µg/mL) indicated no significant trend towards enhancing inflammatory expression at these levels. Additionally, CD206 expression in the ADMH group showed no notable difference from the untreated control, whereas CD206 expression in the groups treated with Ag@ADMH and low-concentration silver nanoparticles was significantly elevated, suggesting that phenolic hydroxyl components within the nanoparticles could enhance the expression of anti-inflammatory factors. Flow cytometry analysis (Fig. 7C) corroborated these findings, showing no significant alteration in M1 (CD206-/CD11C+) expression in ADMH post-inflammation induction compared to the untreated control. However, M1 expression in Ag@ADMH was markedly reduced, with the low-concentration group, AgNPs-1 (5 µg/mL), showing no trend towards M1 reduction. Yet, AgNPs-2 (10 µg/mL) displayed a significant decrease in M1 expression. M2 (CD206+/CD11C-) expression in ADMH did not show significant improvement, whereas its expression in Ag@ADMH was significantly enhanced. AgNPs-1 did not exhibit a notable improvement, but AgNPs-2 showed a significant increase. These results highlight Ag@ADMH’s potential in mitigating inflammatory responses and promoting anti-inflammatory factor expression, suggesting its efficacy in pathogen eradication and tissue repair by modulating the macrophage-mediated inflammatory environment (Fig. 7D, E).

Furthermore, immunofluorescence was utilized to examine the impact of ADMH and Ag@ADMH on the polarization of RAW264.7 macrophages following co-culture, specifically focusing on M1 (iNOS) and M2 (CD206) labeled macrophages. The results, as illustrated in Fig. 7G, revealed a pronounced disparity between the M1 and M2 polarization states in the ADMH and Ag@ADMH groups (0.53 ± 0.08 versus 3.039 ± 0.19, *p* < 0.0001). This disparity underscores ADMH’s propensity to induce a pro-inflammatory response, whereas Ag@ADMH exhibits a marked anti-inflammatory trend. These observations collectively indicate that while ADMH influences the expression of pro-inflammatory factors in macrophages, Ag@ADMH significantly modulates macrophage polarization, attenuating pro-inflammatory factor expression and enhancing M2 polarization. Thus, Ag@ADMH demonstrates pronounced immunomodulatory capabilities, fostering a conducive immune microenvironment. The sustained release of silver nanoparticles, enriched with phenolic hydroxyl groups, is posited as a key factor underpinning the effective immunomodulatory action of Ag@ADMH, offering promising implications for its application in immunomodulation and tissue repair strategies.

### Mouse subcutaneous balloon model

**Fig. 8:**
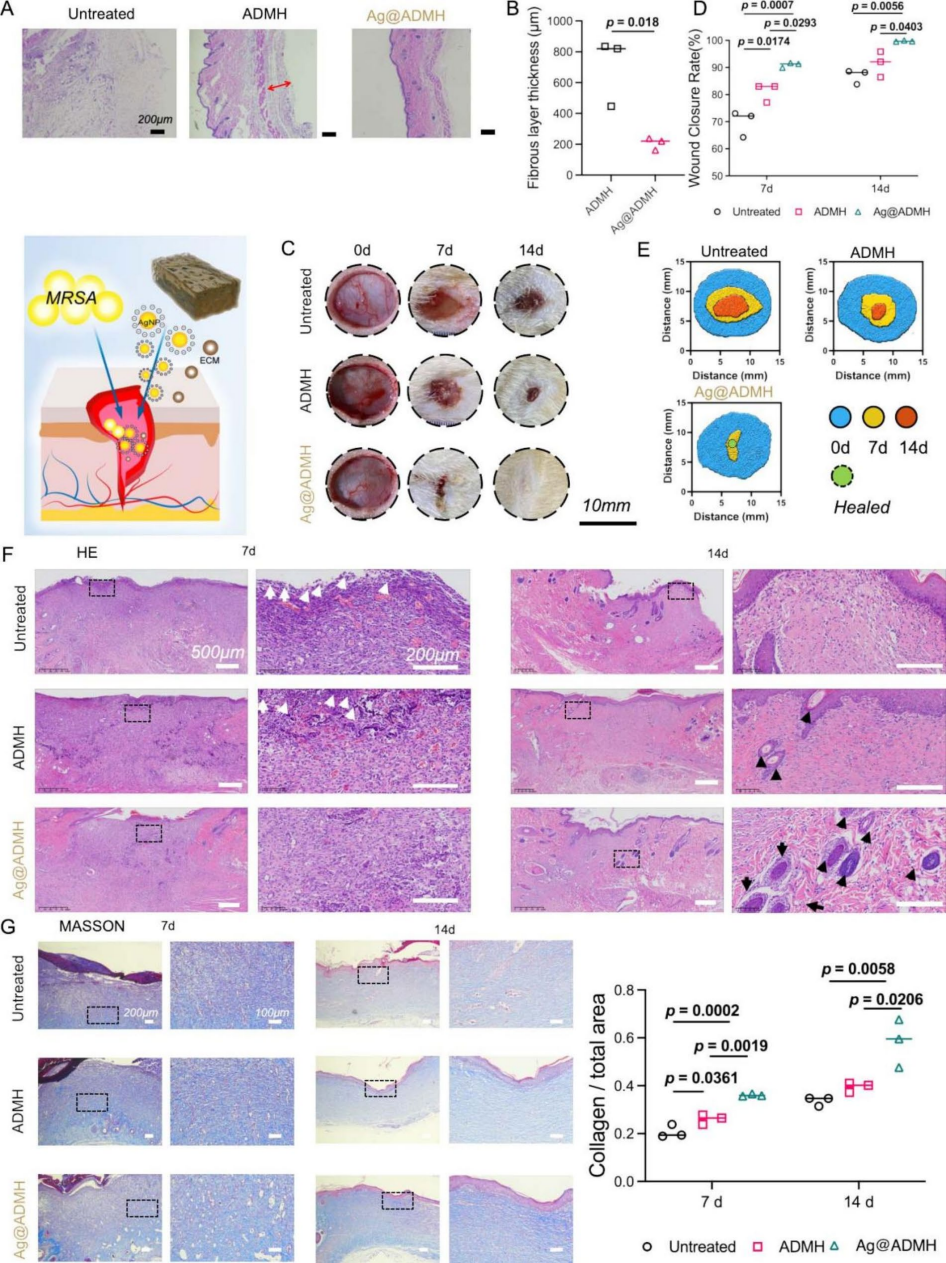
**A**,** B** H&E staining and quantitative analysis of subcutaneous airbag thickness experiment. **C-E** Healing trend analysis of skin defect experiment infected with MRSA. **F** H&E staining of wound skin tissue on days 7 and 14. White arrows indicate inflammatory cells; black arrows indicate hair follicles. **G** MASSON staining and statistical analysis of collagen deposition in wound skin tissue on days 7 and 14

One week post-subcutaneous implantation, the mice were euthanized, and all samples fully infiltrated by cells were retrieved from the surrounding skin to evaluate the level of inflammation [36]. Hematoxylin and Eosin (H&E) staining of skin sections were employed for this assessment. As depicted in Fig. 8A, B, the blank group exhibited an unclear structure, confirming the successful establishment of the inflammation model. Notably, the thickness of the surrounding fibrous layer was significantly diminished in the Ag@ADMH samples in comparison to those treated with ADMH, suggesting a mitigated inflammatory response. Macrophages, which are pivotal in the body’s response to biomaterials, are drawn to the surface of these materials post-implantation and play a critical role in local chronic inflammation, including their association with foreign body giant cells. This interaction often leads to the formation of fibrous capsules around the biomaterial, a typical reaction to subcutaneous implantation, with the capsule’s thickness being indicative of the implant’s inflammatory impact. Thus, the reduced fibrous layer thickness in Ag@ADMH-treated samples signifies not only a lower inflammatory response but also points to the material’s capacity to modulate macrophage activity and potentially minimize chronic inflammation associated with implantation.

### Wound healing model of MRSA infection in rats

The discussion on the potential of Ag@ADMH to combat bacterial infections and aid in infected wound repair was a critical aspect of our study. We have conducted preliminary in vivo research focusing on the wound healing process in a methicillin-resistant Staphylococcus aureus (MRSA) infected wound model. To evaluate the wound healing efficacy of Ag@ADMH, a full-thickness skin defect model was utilized. Following anesthesia, a ∼1.2 cm diameter tissue punch was used to create a wound on the back skin, which was then infected with MRSA for several hours before applying the hydrogel treatment. The untreated wound served as a control. Applied directly to the wound site, the hydrogel functions as a wound dressing, filling tissue gaps and serving as a scaffold for in-situ tissue engineering. As presented in Fig. 8C-E, MRSA-infected wounds without any intervention struggled to heal fully, with only 89.47% of wound areas closing after 14 days. This closure rate contrasts with approximately 94% in the ADMH hydrogel-treated group, attributed in part to the natural contraction of rat skin wounds. However, the wound closure rate in the Ag@ADMH hydrogel group surpassed 99%, significantly outperforming the other groups, with minimal scarring observed. Over the course of 7 and 14 days, we employed a range of histological and immunohistochemical analyses to evaluate the healing efficacy of the Ag@ADMH hydrogel. Specifically, H&E staining was used to assess general tissue morphology, while Masson’s trichrome staining provided insights into collagen deposition and the overall structural integrity of the healing tissue [37].In Fig. 8F&G, on day 7, untreated wounds exhibited a higher presence of inflammatory cells (indicated by white arrows) and a scarcity of collagen fibers. In contrast, wounds treated with Ag@ADMH hydrogels demonstrated a proliferation of collagen fibers. On day 14, although some collagen fibers were visible in untreated wounds, they were still plagued by numerous inflammatory cells. Meanwhile, the ADMH-treated wounds featured a thin epithelium and few hair follicles (denoted by black arrows). Wounds treated with Ag@ADMH hydrogels, however, exhibited a well-organized epidermis, an abundance of hair follicles, and dense collagen formation. Collagen, a vital component of the extracellular matrix, plays a crucial role in wound healing. It accumulates and cross-links at the wound site, forming collagen fibers that reinforce the wound tissue, thereby promoting the healing process. These findings underscore Ag@ADMH’s potential as an effective treatment for enhancing wound closure, reducing inflammation, and supporting tissue regeneration in infected wounds.

**Fig. 9:**
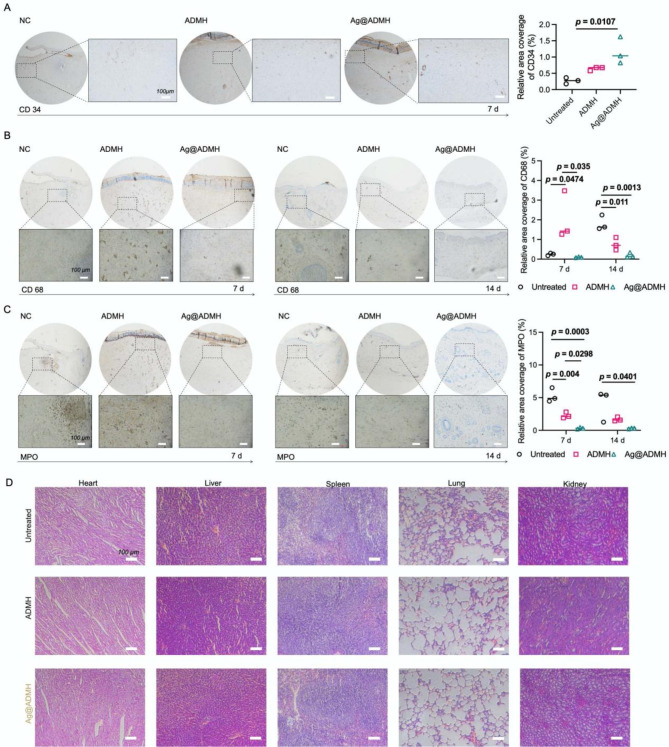
**A-C** Immunohistochemical results and quantitative analysis of CD34, CD68, and MPO in wound skin tissue on days 7 and 14. **D** H&E staining of heart, liver, spleen, lung, and kidney

Additionally, CD34 immunohistochemistry was conducted to quantify angiogenesis, which is crucial for effective wound healing. Prolonged inflammation can inhibit wound repair and lead to scar formation, which is detrimental to skin regeneration [38, 39]. Numerous studies have demonstrated that modulating the inflammatory response in damaged skin is essential for proper wound healing [39, 40]. To evaluate the inflammatory response, MPO staining was utilized to track neutrophil infiltration, and CD68 staining was used to assess macrophage presence and activity [41–43]. Immunohistochemical staining results, depicted in Fig. 9A, highlighted significant findings. On day 7 post-injury, CD34 expression in the Ag@ADMH hydrogel group was markedly elevated compared to the ADMH and blank groups. This indicates the crucial role of angiogenesis in tissue remodeling, facilitated by the natural ECM structure and the composition of the hydrogel, which promotes blood vessel formation. CD68 labeled macrophages and MPO labeled neutrophils were analyzed to gauge the level of inflammatory response. Seven days post-injury, all groups exhibited varying degrees of inflammation, as evidenced by CD68 expression levels. On day 14, a significant reduction in CD68 expression was observed across all groups, signaling an alleviation of the inflammatory response. Notably, the CD68 expression in the Ag@ADMH hydrogel group was significantly lower than in both the blank and ADMH groups on both days 7 and 14 post-injury, suggesting the anti-inflammatory effects of silver nanoparticles within the hydrogels that might mitigate the inflammatory response in infected wounds (Fig. 9B). On day 7, a high presence of MPO was noted in both the blank and ADMH groups, indicating a severe infection and an acute stage of inflammation. Conversely, the Ag@ADMH group exhibited negligible MPO expression, implying effective infection control. On day 14, the pattern of MPO expression remained consistent, underscoring the ability of the Ag@ADMH group to manage the wound’s inflammatory microenvironment and foster healing (Fig. 9C). Figure 9D presents H&E staining of major rat organs (heart, liver, spleen, lungs, and kidneys), revealing no histopathological changes (such as necrosis or altered cellular structure), attesting to the safety of Ag@ADMH in wound treatment without inducing tissue abnormalities. The results from these analyses demonstrated that Ag@ADMH significantly accelerates wound closure, enhances collagen deposition, and promotes angiogenesis, all while effectively reducing the inflammatory response at the wound site. These findings strongly suggest that Ag@ADMH is not only effective in combating bacterial infections but also plays a pivotal role in enhancing the wound healing process, making it a promising candidate for the treatment of infected wounds.

## Conclusion

This investigation led to the development of an injectable hydrogel designed to effectively fill irregular wounds. By integrating plant polyphenols, known for their capacity to modulate macrophage polarization, with silver nanoparticles, renowned for their anti-inflammatory properties, into a dermal extracellular matrix (ECM) hydrogel, this novel formulation aims to address the issue of excessive M1 polarization associated with standard dermal ECM hydrogels. Ag@ADMH stands out for its ability to regulate the inflammatory immune microenvironment, encouraging the shift towards an M2 macrophage phenotype, which is crucial for the healing of chronic wounds. The study’s findings confirm that Ag@ADMH exhibits significant anti-inflammatory effects, enhances the expression of the vascular marker CD34, and reduces the levels of CD68 and MPO, markers of inflammation. Additionally, it facilitates collagen deposition, a critical factor in tissue repair and wound healing. These attributes collectively suggest that Ag@ADMH hydrogels hold considerable promise for clinical application in the management of chronic wounds, offering a multifaceted approach to wound care that encompasses inflammation modulation, angiogenesis promotion, and enhanced tissue regeneration.

## Electronic supplementary material

Below is the link to the electronic supplementary material.

Supplementary Material 1

## Data Availability

The datasets used and/or analyzed during the current study are available from the corresponding author on reasonable request.

## Abbreviations

GA: Gallic acid
Ag NPs: Silver nanoparticles
SPM: Spread Plate Method
ADMH: Acellular Dermal Matrix hydrogel
Ag@ADMH: AgNPs- acellular dermal matrix hydrogel
MRSA: Methicillin-resistant Staphylococcus aureus
DPPH: 2,2-diphenyl-1-picrylhydrazyl
SEM: Scanning electron microscope
TEM: Transmission Electron Microscopy
