# Supplementary material for: Designing injectable dermal matrix hydrogel combined with silver nanoparticles for methicillin-resistant Staphylococcus aureus infected wounds healing
Source: Nano Converg. 2024 Oct 17;11:41. doi: 10.1186/s40580-024-00447-0 (PMC11486888; doi:10.1186/s40580-024-00447-0)
Supplement: Supplementary file 1 — Supplementary Material 1 [file 40580_2024_447_MOESM1_ESM.docx]

**Supporting Information**

**Designing injectable dermal matrix hydrogel combined with silver nanoparticles for methicillin-resistant Staphylococcus aureus infected wounds healing**

Sunfang Chen^1#^, Jun Yao^1#^, Shicheng Huo^2#^, Chennan Xu^1^, Ruting Yang^1^, Danhua Tao^3^, Bin Fang^1^, Gaoxiang Ma^1^, Zaihua Zhu^4*^, Ye Zhang^1*^, JingJing Guo^5*^

1 Department of Orthopedic Surgery, Spine Center, the Central Hospital Affiliated to Shaoxing University, Shaoxing, 321030, China

2 Department of Orthopedic Surgery, Spine Center, Changzheng Hospital, Navy Medical University, Shanghai, 200003, China

3 Department of Pathology, the Central Hospital Affiliated to Shaoxing University, Shaoxing, 321030, China

4 Division of Rheumatology and Immunology, Huashan Hospital, Fudan University, Shanghai, 200040, China.

5 Department of Pharmacy, the Central Hospital Affiliated to Shaoxing University, Shaoxing, 321030, China

^#^These authors contribute equally.

* Indicates the corresponding author

* Corresponding author: Zaihua Zhu, Ye Zhang, JingJing Guo

E-mail address: [9094116@qq.com(Z,Zh)](mailto:9094116@qq.com(Z,Zh)), [809144858@qq.com(Y,Zh)](http://www.809144858@qq.com(Y,Zh)), guojingjing-www@163.com(J,G)

1. **Supplementary Experimental Section**
   1. **Hydrogel degradation *in vitro***

To assess the degradation of ADMH and Ag@ADMH at pH 5 and 7.4[1], 500μg of each hydrogel were allocated into separate centrifuge tubes. Periodic inspections were conducted to monitor for any fluid secretion or complete disintegration of the hydrogels. Post-degradation, the hydrogel components were extracted, and the residual weight was quantified to evaluate the extent of degradation.

- 1. **AgNPs releasing performance of Ag@ADMH**

To investigate the effect of pH on the release kinetics of silver nanoparticles (AgNPs) from Ag@ADMH, a 500 μg sample was distributed in centrifuge tubes, each containing 1 mL of PBS solution. The samples were then incubated at two different pH levels: 5 and 7.4, simulating the pH conditions of infection and normal physiological states [1], respectively, and maintained at 37°C to mimic body temperature. The release of silver nanoparticles into the PBS solution was monitored at various time intervals to evaluate how changes in pH influence the diffusion rate and release mechanisms of AgNPs from the hydrogel matrix. This approach provides a comprehensive understanding of the pH-dependent release kinetics of AgNPs, which is critical for optimizing therapeutic efficacy and safety in clinical applications.

1. **Supplementary Figure**


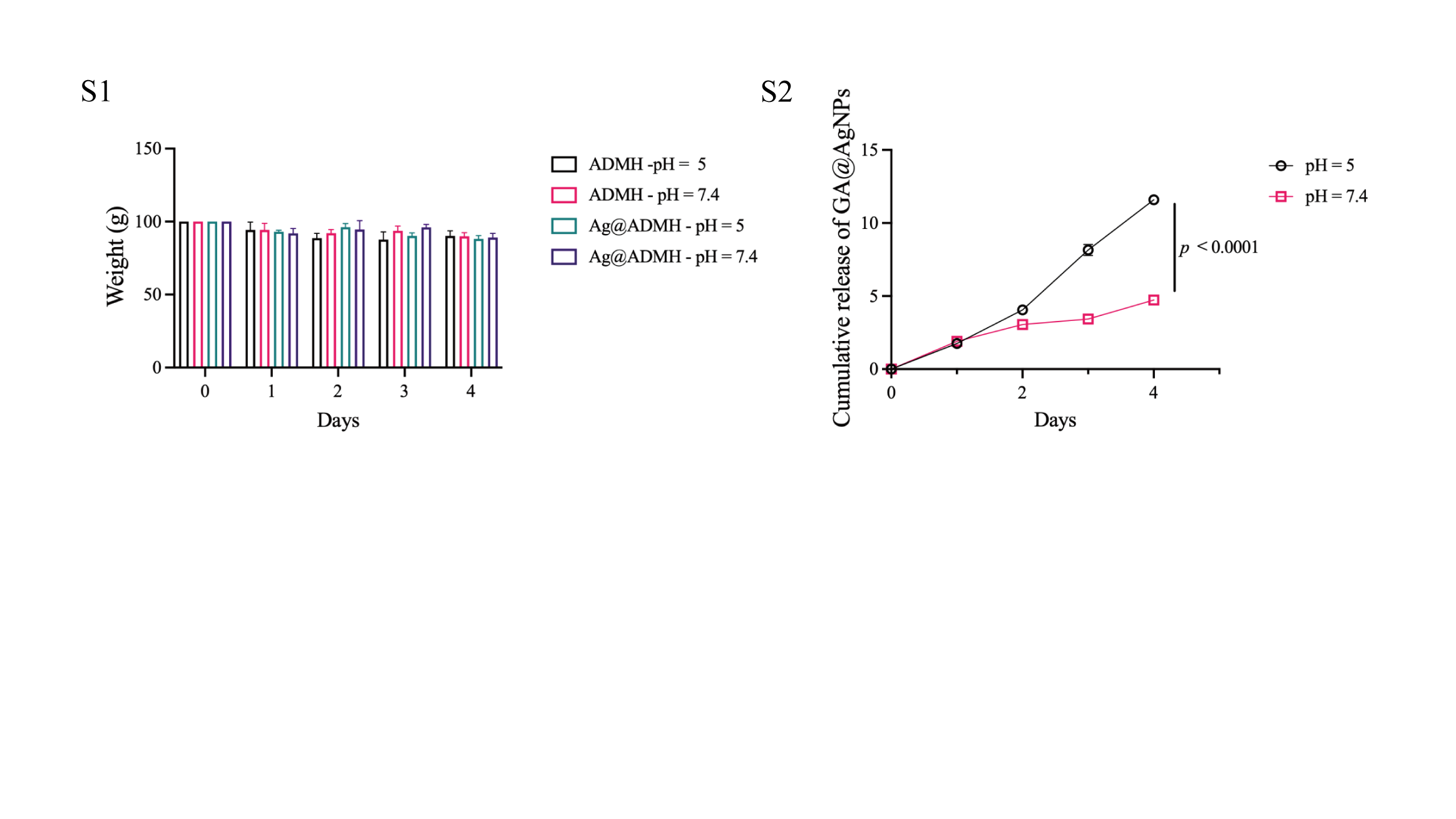
 **S1**: Degradation of ADMH and Ag@ADMH hydrogel（*p* > 0.05)；**S2**: Cumulative release of GA@AgNPs from Ag@ADMH（*p* < 0.0001).

**References**

1. Fang, B., et al., *Extracellular matrix scaffold crosslinked with vancomycin for multifunctional antibacterial bone infection therapy.* Biomaterials, 2021. **268**: p. 120603.
